# Altered microvascular density in patients with systemic lupus erythematosus treated with hydroxychloroquine—an optical coherence tomography angiography study

**DOI:** 10.1007/s00417-020-04788-4

**Published:** 2020-06-12

**Authors:** Nataša Mihailovic, Martin Dominik Leclaire, Nicole Eter, Viktoria C. Brücher

**Affiliations:** Department of Ophthalmology, University of Muenster Medical Center, Albert-Schweitzer-Campus 1, Building D15, 48149 Muenster, Germany

**Keywords:** SLE, HCQ, Vessel density, Choriocapillaris, Foveal avascular zone

## Abstract

**Purpose:**

To evaluate the retinal microvascular density using optical coherence tomography angiography (OCTA) in patients with systemic lupus erythematosus (SLE) treated with hydroxychloroquine (HCQ).

**Methods:**

Nineteen eyes of 19 patients with SLE (study group) without HCQ retinopathy and 19 eyes of 19 healthy subjects (control group) were included in this study. The study group was divided into patients using HCQ for > 5 years (high-risk group) and < 5 years (low-risk group). The VD data of the 3 × 3 mm OCT angiogram of the superficial capillary plexus (SCP) and the choriocapillaris (VD-CC), the foveal avascular zone (FAZ) area and the central retinal thickness (CRT) were extracted and analyzed.

**Results:**

VD in the *en face* SCP was significantly reduced in the high-risk group and the low-risk group compared with that in the control group (*p* < 0.001, *p* = 0.001) and in the high-risk group compared with the low-risk group (*p* = 0.007). Correlation analysis between the cumulative dose of HCQ and the VD of the study group revealed a negative correlation, but no statistical significance (*p* = 0.074). However, a significant positive correlation was observed for the low-risk group (*p* = 0.035). In patients with SLE, VD-CC was lower (*p* = 0.042) and the FAZ area larger (*p* = 0.019). CRT showed no difference between the groups (*p* = 0.183).

**Conclusion:**

In this study, SLE patients showed a reduced VD in both groups. In patients treated with HCQ < 5 years, HCQ might have a protective effect on retinal microvasculature. Analysis of retinal microvascular density using OCTA could be useful in the diagnosis and monitoring of vascular alteration in patients with SLE.

## Introduction

Systemic lupus erythematosus (SLE) is an inflammatory autoimmune connective tissue disease with heterogenous disease expression and variable course of disease. Involvement of a variety of organs might occur either due to direct autoimmune processes or due to secondary pathologic states like hypercoagulability and vasculitis [1]. Ocular involvement particularly includes external and anterior segment diseases, especially dry eye syndrome, as well as posterior segment diseases like optic neuritis, ischemic optic neuropathy, retinal vasculitis, occlusive retinopathies, and choroidopathies [2]. Detection of posterior segment involvement is crucial for both evaluating the disease activity and preventing permanent visual loss. An approved treatment for SLE is hydroxychloroquine (HCQ) which might cause toxic retinopathy itself. The risk of toxicity at recommended doses up to 5 years of treatment is < 1 %, but it increases sharply after approximately 5 years of therapy [3]. Early detection of HCQ retinopathy is important, since its damage is irreversible and can progress even after cessation of the therapy [3].

In addition to indirect ophthalmoscopy, retinal and choroidal vascular alterations due to microangiopathy in SLE have most commonly been visualized using fluorescein angiography (FA) and indocyanine green angiography (ICG) [4, 5]. Both, FA and ICG are dye-based and invasive imaging techniques, unlike optical coherence tomography angiography (OCTA). With OCTA, an innovative imaging modality, chorioretinal vasculature can be visualized in a non-invasive and dyeless way. OCTA provides a three-dimensional visualization of choroidal and retinal microvascular layers as well as the possibility of quantification of the vessel density, which up to now has not been possible with FA and ICG [6]. Different study groups demonstrated an impaired VD of the superficial retinal capillary plexus (SCP) in patients with SLE using OCTA [7–9]. Additionally, Conigliaro et al. showed a positive correlation between the cumulative dose of HCQ and VD in their patients [9]. These studies, however, either included only patients with a duration of HCQ treatment for > 5 years, did not differentiate between patients treated with HCQ for more and less than 5 years or also included patients with other connective tissue diseases like rheumatoid arthritis. Also, to the best of our knowledge, a VD analysis of the OCT angiogram of the choriocapillaris (VD-CC) in patients with SLE has not been described in the literature yet.

The primary objectives of the present OCTA study were:

1. To evaluate VD differences between healthy controls, patients with SLE and a short use of HCQ (< 5 years) and patients with SLE and a long use of HCQ (> 5 years)
2. To detect a possible relation between VD and the cumulative dose of HCQ.

Secondary objectives were the quantitative analysis of the VD-CC, foveal avascular zone (FAZ), and central retinal thickness (CRT).

## Methods

Nineteen eyes of 19 patients with SLE and 19 eyes of 19 gender- and age-matched healthy controls were consecutively included in this study. All analysis of parameters was performed using the data of the right eye in both, patients and controls. The study was approved by the Ethics Committee of the University of Muenster, North Rhine Westphalia, Germany. Before performing any examination, the study protocol was explained in detail and all participants signed an informed consent form. The study adhered to the tenets of the Declaration of Helsinki. Patients and controls with media opacities preventing high-quality imaging, vitreoretinal disease, previous retinal surgery, macular edema, glaucoma, or neurological disease were excluded from the study. All study participants underwent an ophthalmic examination including anterior segment examination, binocular fundus examination, and OCTA imaging. In the patient group, 10-2 visual field testing (standard automated perimetry), multifocal electroretinogram, fundus autofluorescence, and spectral-domain OCT scanning of the macula and optic nerve head (RNFL) using the Spectralis OCT (Heidelberg Engineering, Germany) was performed to rule out SLE-associated retinopathy or HCQ toxicity. Only patients with no signs of retinopathy or HCQ toxicity were included.

Patients treated with HCQ for > 5 years were classified as the “high-risk group” (*n* = 9), and patients with a HCQ treatment for < 5 years were classified as the “low-risk group” (*n* = 10). This classification was based on the revised recommendations on screening for HCQ therapy of the American Academy of Ophthalmology which define a duration of use > 5 years as a factor of increasing risk of HCQ retinopathy [3]. For the correlation analysis of the VD and the cumulative dose of HCQ, cumulative dose data could be extracted from all patient records except from one (*n* = 18).

### Optical coherence tomography angiography

Optical coherence tomography angiography (OCTA) imaging was performed using the RTVue XR Avanti system with AngioVue (Optovue Inc, Fremont, California, USA). Split-spectrum amplitude-decorrelation angiography (SSADA) was used to extract the OCTA information. OCTA imaging technology has been described in detail elsewhere [6]. The macula was imaged using a 3 × 3 mm scan. The automated segmentation was checked by an expert examiner before data analysis. All examinations were performed under the same conditions at the same location by an expert examiner. Images with poor signal strength (signal strength index < 6) were not included in the study. After imaging, the FAZ area and the VD in SCP OCT angiogram were analyzed using the integrated device software (AngioAnalytics, version 2017.1.0.151, Optovue Inc, Fremont, California, USA). To determine the VD values of the choriocapillaris, OCTA images were exported. Using Adobe Photoshop CS6 (Adobe Systems, Inc., California), images were converted into grey scales. Each pixel was attributed to a value that represents the strength of the decorrelation signal. VD in the choriocapillaris was calculated as the mean decorrelation value of all pixels in the images (arbitrary unit, AU) [10].

### Statistics

IBM SPSS® Statistics 22 for Windows (IBM Corporation, Somers, NY, USA) and GraphPad Prism 5 (GraphPad Software Inc., San Diego, CA, USA) were used for statistical analyses. The data was tested for normality distribution using the Shapiro–Wilk test and all data did fit a normal distribution. The data are therefore presented as mean value ± standard deviation. For measurement of effect size, we used Hedges g.

A closed testing procedure was used for the main objective evaluating VD differences between the three groups: low-risk group, high-risk group, and control group. Specifically, a one-way analysis of variance (ANOVA) was performed followed by a post hoc analysis via two-sided independent sample *t* tests. The degree of correlation between VD and the cumulative dose of HCQ was expressed as the Pearson correlation coefficient (PCC). The chosen level of statistical significance was *p* < 0.05. The secondary objective analysis was exploratory, not confirmatory, and the data were compared using two-sided independent sample *t* tests.

## Results

There was no significant difference in age between SLE patients and healthy controls (*p* = 0.630) and between patients of the high-risk group and the low-risk group (*p* = 0.857). The demographic characteristics of patients and healthy controls are shown in Table 1. For the comparisons of HCQ therapy and cumulative doses between the low-risk group and the high-risk group, the values of Hedges g were 1.75 and 1.86, which puts the data into the very large to huge effect sizes. It is about the equivalent of ROC AUCs of 0.893 and 0.905 [11].

**Table 1:** Demographic data of study population

	Control group	Study group	*p* value	Low-risk group	High-risk group	*p* value
*n*	19	19	-	10	9	-
Gender f/m	14/5	14/5	-	8/2	6/3	-
Mean age (years) ± SD	38.2 ± 12.6	40.1 ± 11.5	0.630	40.4 ± 10.5	39.7 ± 13.2	0.896
mean duration of HCQ therapy (years) ± SD	-	5.76 ± 5.18	-	2.71 ± 2.68	9.46 ± 4.55	< 0.001
mean cumulative dose of (g) ± SD	-	819 ± 773	-	265 ± 218	1317 ± 754	0.002

*Low-risk group*, SLE patients treated with HCQ < 5 years; *High-risk group*, SLE patients treated with HCQ >5 years. *SD*, standard deviation; *HCQ*, hydroxychloroquine; *g*, gram; *f*, female; *m*, male

**Fig. 1:**
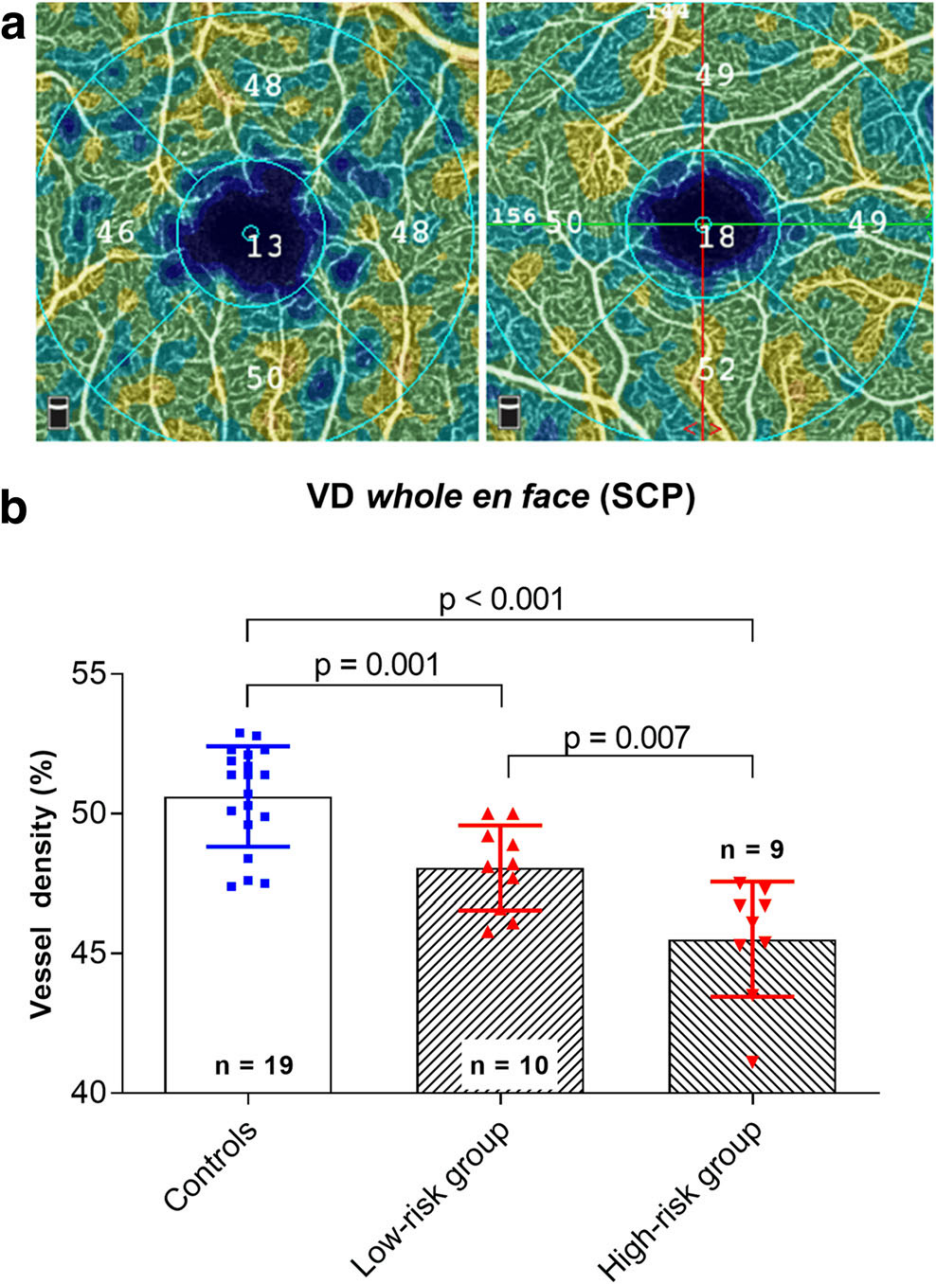
**a** Exemplary color-coded superficial capillary plexus (SCP) *whole en face* optical coherence tomography (OCT) angiogram (3 × 3 mm) of a patient with systemic lupus erythematosus (SLE) (left) and a healthy control (right). **b** Comparison of the vessel density (VD) of the SCP *whole en face* OCT angiogram between controls and patients treated with hydroxychloroquine (HCQ) for < 5 years (low-risk group) and > 5 years (high-risk group) as well as between the low-risk group and high-risk group

One-way-ANOVA revealed a significant difference between the means for VD in the SCP OCT angiogram (*whole en face*) of the control group, the high-risk group, and the low-risk group (*p* < 0.001). Post hoc analysis showed a significantly reduced VD in the high-risk group and the low-risk group compared with that in the control group (VD controls, 50.62 ± 1.80; VD high-risk, 45.51 ± 2.06, *p* < 0.001; VD controls, 50.62 ± 1.80; VD low-risk, 48.06 ± 1.52, *p* = 0.001). Moreover, a significantly lower VD was found in the high-risk group compared with that in the low-risk group (*p* = 0.007; Fig. 1a, b).

**Fig. 2:**
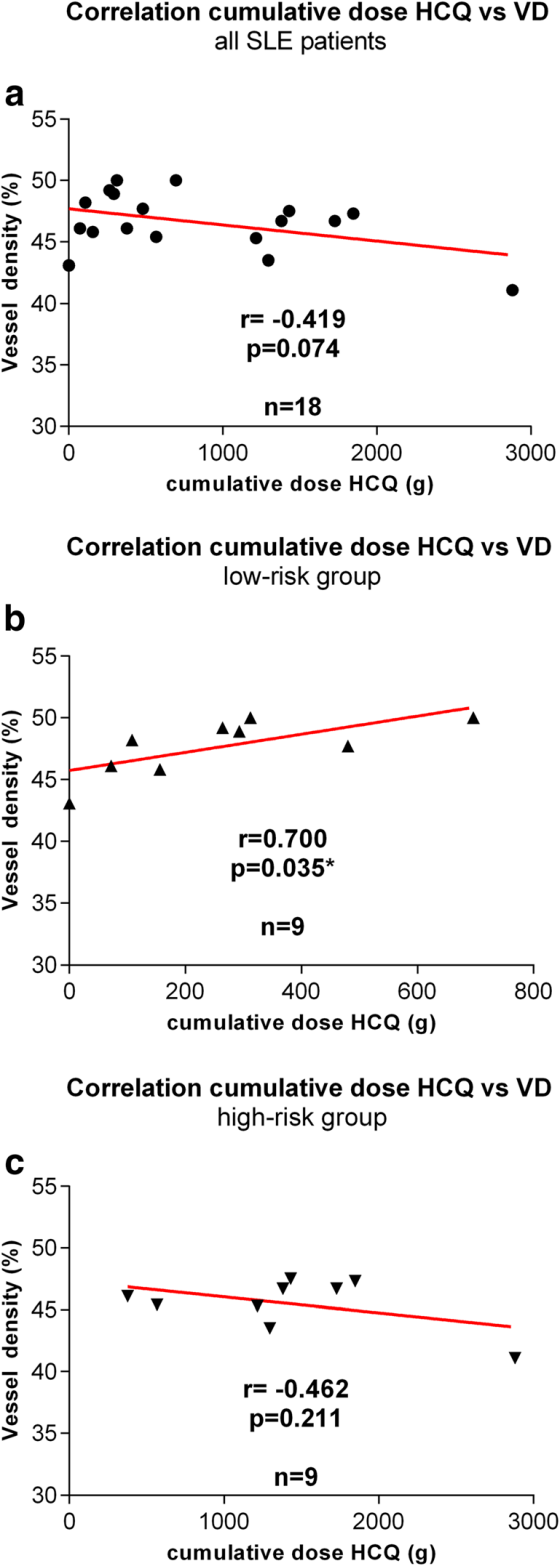
Correlation analysis of the vessel density (VD) and the cumulative dose of hydroxychloroquine (HCQ) for all patients (a), patients treated with hydroxychloroquine (HCQ) for < 5 years (low-risk group, b) and > 5 years (high-risk group, c) (*r* = Pearson correlation coefficient, * = *p* < 0.05, red line = linear regression)

The correlation analysis of the VD and the cumulative dose of HCQ indicated an overall negative correlation for the whole study group (PCC − 0.419, *p* = 0.074; Fig. 2a). In the low-risk group, however, there was a significantly positive correlation between VD and the cumulative dose of HCQ (PCC 0.700, *p* = 0.035; Fig. 2b). In the high-risk group, no significant correlation was found (PCC − 0.462, *p* = 0.211; Fig. 2c).

**Fig. 3:**
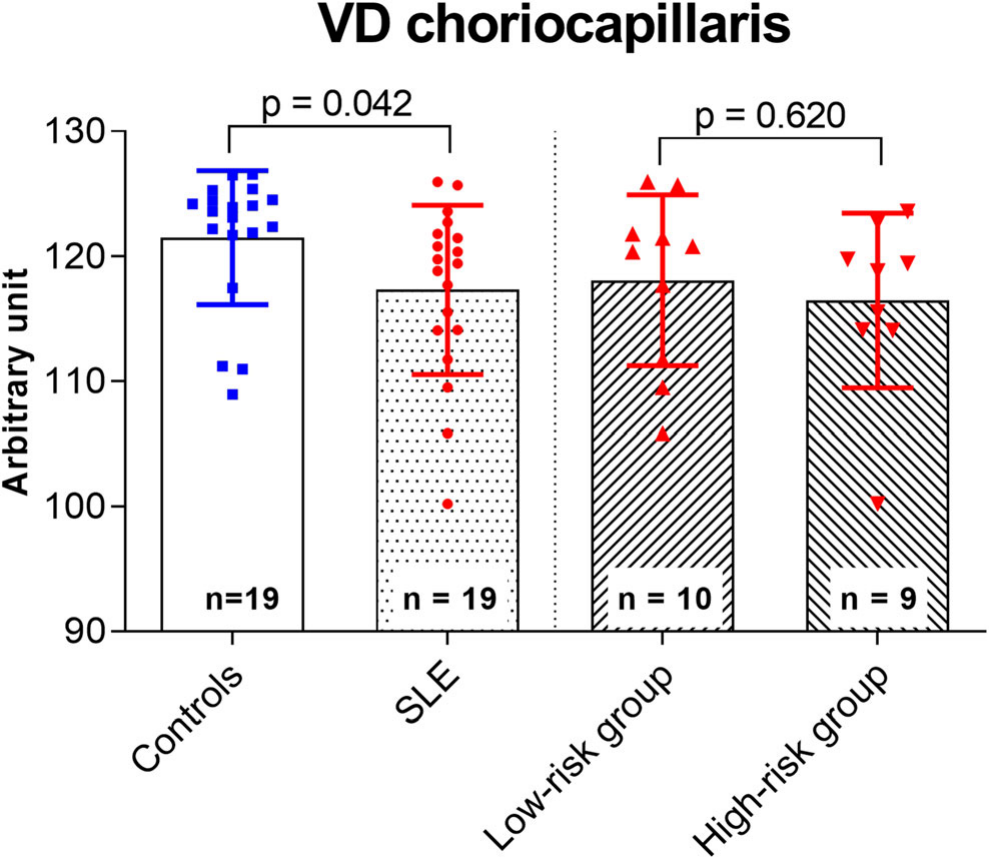
Comparison of the vessel density (VD) of the optical coherence tomography (OCT) angiogram of the choriocapillaris between the study and control group as well as between patients treated with hydroxychloroquine (HCQ) for < 5 years (low-risk group) and > 5 years (high-risk group).

**Fig. 4:**
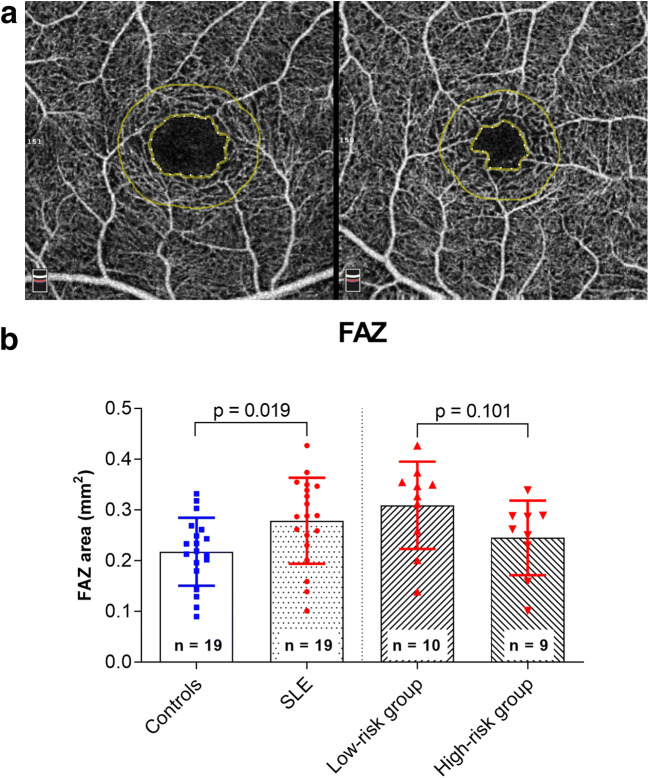
**a** Exemplary color-coded superficial capillary plexus optical coherence tomography (OCT) angiogram (3 × 3 mm) showing the foveal avascular zone (FAZ) measurements of a patient with systemic lupus erythematosus (SLE) treated with hydroxychloroquine (HCQ) for 2 months (left) and a healthy control (right). **b** Comparison of the FAZ area between the study and control group as well as between patients treated with HCQ for < 5 years (low-risk group) and > 5 years (high-risk group)

Choriocapillaris analysis showed a reduced VD in SLE patients compared with that in the healthy controls (study group, 117.34 AU ± 6.78 AU; control group, 121.51 AU ± 5.37 AU, *p* = 0.042; Fig. 3). Additionally, the FAZ area of the SLE patients was larger than the FAZ area of the healthy controls (study group, 0.279 mm^2^ ± 0.085 mm^2^; control group, 0.218 mm^2^ ± 0.067 mm^2^, *p* = 0.019; Fig. 4a, b). Central retinal thickness (CRT) did not differ between SLE patients and healthy controls (study group, 252.4 μm ± 21.3 μm; control group, 263.1 μm ± 16.5 μm, *p* = 0.092).

## Discussion

Microvascular alteration including occlusive vasculopathy in patients with SLE is an important issue of the disease and a major cause for morbidity and mortality in these patients [12, 13]. Involvement of the posterior segment of the eye usually comes along with poor visual outcomes and is indicative of high disease activity [14]. Therefore, the visualization and evaluation of the microvasculature of patients with SLE are of high interest for both, confirming the diagnosis and assessing the disease activity in follow-up examinations. In previous studies, these microvascular alterations have been evaluated using capillaroscopy, infrared thermography, and iontophoresis. For evaluation of retinal and choroidal evaluation, fluorescein and indocyanine green angiography have been used mostly to date [4, 5]. However, these methods are invasive and time-consuming and carry the risk of an anaphylactic reaction [15, 16]. In contrast to that, OCTA is a fast and non-invasive method to visualize vascular alteration in small vessels and capillaries of the retina and choriocapillaris [6, 17].

The results of this OCTA study demonstrate a highly significant reduction of the VD in the SCP OCT angiogram in SLE patients compared with that in a healthy control group. Likewise, findings have been shown in the literature before [7–9]. Contrary to our study, Forte et al. only included patients treated with HCQ for more than 5 years and Conigliaro et al. included an inhomogeneous study population meaning not all of the patients were treated with HCQ and they did not differentiate between long and short treatment with HCQ or the cumulative dose level [8, 9]. Bulut et al. stated that an HCQ therapy of more than 5 years in patients with different underlying rheumatic diseases results in a significantly lower VD and FAZ compared with patients with an HCQ therapy of less than 5 years [18]. However, it cannot be concluded from these findings whether the observed retinal microvascular alterations were genuinely caused by the underlying disease or if these changes could be influenced or biased by the HCQ therapy. Therefore, we did not only focus on the differences between SLE patients and healthy controls in general but also analyzed the differences between patients treated with HCQ for more and less than 5 years. The prevalence of HCQ-induced toxic retinopathy rises sharply after 5 years of therapy [19], which is also reflected in the revised recommendations of the American Academy of Ophthalmology on screening for chloroquine and HCQ retinopathy [3].

Our results demonstrate that even patients with a low-risk profile for HCQ-induced toxic retinopathy (HCQ therapy < 5 years; mean ± SD, 2.71 ± 2.68 years) show a significantly lower VD than healthy controls measured by OCTA. This may suggest that SLE itself leads to a reduced VD in affected patients, regardless of HCQ therapy. Furthermore, there was a significantly reduced VD in patients with a high-risk profile compared with patients with a low-risk profile. This finding supports the assumption that microvascular changes in patients with HCQ use > 5 years significantly differ from patients with HCQ use < 5 years. Nevertheless, this might also be due to the longer course of the disease. However, studies with SLE patients not using HCQ would be more meaningful, but since HCQ is the most usual treatment for patients with SLE, the number of patients without HCQ therapy is very low and a reduced VD in SLE patients not using HCQ has not been evaluated in the literature yet.

The correlation analysis of the VD and the cumulative dose of HCQ indicated an overall negative correlation for the whole study group, but did not reach the chosen level of statistical significance (*p* = 0.074, Fig. 2a). In the low-risk group, however, there was a statistically significant positive correlation between VD and the cumulative dose of HCQ (*p* = 0.035, Fig. 2b). We therefore hypothesize that there might be a protective effect of HCQ leading to a reduced disease activity only in the early stage of the disease. Conigliaro et al. also assumed a protective effect of the cumulative HCQ doses on VD in patients with SLE in general [9]. However, they did not distinguish between patients with different durations of HCQ treatment.

VD-CC in the study group was lower compared with that in the control group (Fig. 3). As indicated by immunofluorescence and electron microscopy, immune complexes accumulate in the choriocapillaris [20] and choroidopathy is one of the possible ophthalmic manifestations in patients with SLE [21]. Choroidal thickness appears to be thinner in SLE patients compared with that in healthy controls using spectral-domain OCT [22]. In patients with HCQ retinopathy, Ahn et al. demonstrated signal void areas on the choriocapillaris in the areas of retinal pigment epithelium defects using OCTA [23]. To the best of our knowledge, a choriocapillaris analysis in SLE patients without HCQ retinopathy using OCTA has not been reported in the literature before. Our findings may lead to the hypothesis that VD-CC can be affected even in patients with SLE without retinopathy. OCTA may help in disclosing these early changes.

FAZ area was enlarged in patients with SLE (Fig. 4). The size of the FAZ alters in different retinal and systemic diseases, which also may correlate with visual function [24]. Bulut et al. also showed a wider FAZ area in patients using HCQ for > 5 years [7, 18]. However, long-term studies evaluating the diagnostic value of these changes in the course of the disease are currently lacking.

The present study has some limitations worth noting. First, we analyzed a small cohort. It might be that some of the negative findings (i.e., lack of significant differences) were due to the limited sample size. However, correcting for small cohort size with Hedges g still revealed large effect sizes. Moreover, the SLE patients described herein account a rather homogeneous study population, since all patients were treated with HCQ and none of the patients showed any signs of ocular involvement. Second, since our study has a cross-sectional design, we cannot comment on the value of VD measurements for evaluation of disease progression. Further studies with a longitudinal design are therefore warranted.

In conclusion, the results of our study show a significant reduction of microvascular density in patients with SLE regardless of the duration of HCQ therapy. Moreover, patients treated with HCQ for more than 5 years displayed a significantly lower VD than those treated with HCQ for less than 5 years. In an early stage of the disease, HCQ seems to have a protective effect on the VD. OCTA-driven VD evaluation could be a supporting tool in the assessment of altered retinal microvasculature in patients with SLE treated with HCQ. However, the exact mechanisms that lead to these findings are not fully understood yet, and further studies are needed to underline the clinical utility of OCTA technology and to elaborate the clinical consequences of these microvascular changes in these patients.

## Data Availability

All data of this study are available from the corresponding author upon reasonable request.
